# Factors affecting early identification of pregnant women by community health workers in Morogoro, Tanzania

**DOI:** 10.1186/s12889-019-7179-1

**Published:** 2019-07-08

**Authors:** G. Frumence, M. Goodman, J. J. Chebet, I. Mosha, D. Bishanga, D. Chitama, P. J. Winch, J. Killewo, A. H. Baqui

**Affiliations:** 1 Department of Development Studies, School of Public Health and Social Sciences, Muhimbili University of Health and Allied Sciences, P.O Box 65454, Dar es Salaam, Tanzania; 2 Department of International Health, Johns Hopkins Bloomberg School of Public Health, 615 N. Wolfe Street, Baltimore, MD USA; 3 Department of Health Promotion Sciences, Mel and Enid Zuckerman College of Public Health, University of Arizona, 1295 N. Martin Avenue, Tucson, AZ 85724 USA; 4 Department of Behavioral Sciences, School of Public Health and Social Sciences, Muhimbili University of Health and Allied Sciences, P.O Box 65015, Dar es Salaam, Tanzania; 5 Jhpiego Tanzania, PO Box 9170, Dar es Salaam, Tanzania; 6 Department of Biostatistics and Epidemiology, Muhimbili University of Health and Allied Sciences, Box 65015, Dar es Salaam, PO Tanzania

**Keywords:** Community health workers, Pregnancy identification, Antenatal care, Trust

## Abstract

**Background:**

It is recommended that Antenatal Care (ANC) be initiated within the first trimester of pregnancy for essential interventions, such folic acid supplementation, to be effective. In Tanzania, only 24% of mothers attend their first ANC appointment during their first trimester. Studies have shown that women who have had contact with a health worker are more likely to attend their first antenatal care appointment earlier in pregnancy. Community health workers (CHWs) are in an opportune position to be this contact. This study explored CHW experiences with identifying women early in gestation to refer them to facility-based antenatal care services in Morogoro, Tanzania.

**Methods:**

This qualitative study employed 10 semi-structured focus group discussions, 5 with 34 CHWs and 5 with 34 recently delivered women in three districts in Morogoro, Tanzania. A thematic analytical approach was used to identify emerging themes among the CHW and RDW responses.

**Results:**

Study findings show CHWs play a major role in identifying pregnant women in their communities and linking them with health facilities. Lack of trust and other factors, however, affect early pregnancy identification by the CHWs. They utilize several methods to identify pregnant women, including: asking direct questions to households when collecting information on the national census, conducting frequent household visits and getting information about pregnant women from health facilities.

**Conclusions:**

We present a framework for the interaction of factors that affect CHWs’ ability to identify pregnant women early in gestation. Further studies need to be conducted investigating optimal workload for CHWs, as well as reasons pregnant women might conceal their pregnancies.

**Electronic supplementary material:**

The online version of this article (10.1186/s12889-019-7179-1) contains supplementary material, which is available to authorized users.

## Background

In 2008, the Tanzanian Ministry of Health, Community Development, Gender, Elderly, and Children (MoHCDGEC) outlined a strategic plan to scale up integrated community-based maternal, newborn, and child health services in remote and underserved areas. The plan includes the provision of trainings for community health workers (CHWs) to deliver health education, including antenatal care (ANC) referrals to new mothers [1–3]. The plan requires CHWs to reach new mothers early in pregnancy, ideally in their first trimester, in order to provide referrals to ANC services.

Antenatal folate supplements must be taken in the first six weeks of pregnancy in order to allow time for them to prevent neural tube defects in newborns. Iron supplementation must also begin early in pregnancy to maintain the proper levels of iron [4]. If women attend their first ANC visit later in pregnancy, there may not be sufficient time for them to receive the full doses of vaccines, treatments, and micronutrients to prevent pregnancy complications. Furthermore, receiving ANC early in the course of pregnancy allows for the identification and management of complications that can lead to high risk births such as, gestational diabetes, HIV, STDs, and anemia [5].

The 2015–2016 Demographic and Health Survey from Tanzania showed that 51% of mothers attended all four recommended ANC visits [6]. Twenty four percent of mothers attended their first ANC appointment during their first trimester, while 33.9% of mothers attended their first ANC visit during their third trimester [6]. Another study found that 80% of women in Tanzania don’t attend their first ANC appointment until their second trimester [7]. Women with unintended pregnancies, and those who are not the head of their household, are more likely to delay their first ANC appointment [8]. By delaying their first ANC appointments, these women miss out on essential preventive services to ensure their health, and the health of their child. Factors such as, maternal level of education, household wealth, level of participation in decision-making, and amount of media exposure have demonstrated a positive association with women attending ANC appointments during their first trimester [9]. Women who have contact with a health worker, moreover, are more likely to attend ANC visits early in their pregnancy [8]. Community health workers are in an opportune position to be this contact, making it important for them to be able to identify pregnant women in their communities early in their pregnancies. The underlying rationales is that the community recognizes that CHWs are working to support pregnant women in their community and therefore, develop a trusting relationship with them [10].

Community health workers often perform home visits in order to identify pregnant women [10]. In fact, the more time the CHW is active in the community, the more success they have identifying pregnant women [10]. In the case of unidentified pregnant women, it may be that women are fully aware that they are pregnant, but wish to conceal it from their social group until later in the pregnancy, or until delivery [11]. According to prior studies, reasons for women, or families of women, to conceal their pregnancies are often socially motivated; typically involving fear of others’ reaction to the pregnancy, and impacted by how far along the pregnancy is [12, 13]. In more traditional societies, fear of social exclusion or ostracism could lead a mother to deny or conceal her pregnancy from her family and or community. Alternatively, concealment of pregnancy could be motivated by fear of abandonment or a fear of losing custody of the child [12]. The idea of “social risk” encompasses these socially-motivated decisions to conceal a pregnancy or not [14].

Concealment of pregnant represents a barrier to the provision of necessary and effective ANC services early in pregnancy, regardless of the reasons for it. According to a Zambian study, women’s interpersonal relationships can have an impact on whether or not she seeks professional care at any point in her pregnancy. These interpersonal relationships extend to CHWs from whom they can also get key information and training to assist with their pregnancy [15]. Characterizing strategies used by CHWs to identify pregnant women will help inform CHW programs in Tanzania, as programs prepare to be scaled-up nationally [16]. This study aimed to explore the attitudes of recently delivered women (RDW) and the experiences of CHWs in identifying women early in their pregnancies in Morogoro, Tanzania.

## Methods

### Study area

This study was conducted as a part of the Morogoro Evaluation Project, which evaluated an integrated maternal, newborn, and child health (MNCH) program (called MAISHA) implemented by the MoHCDGEC in collaboration with the nonprofit global health organization, Jhpiego. Jhpiego, an affiliate of Johns Hopkins University, was founded in 1974 and currently works in over 40 countries with the aim of improving the lives of women and families. LeFevre et al. provides a more detailed description of the larger evaluation [3].

Data were collected in three districts of the Morogoro region: Ulanga, Mvomero and Kilosa/Gairo. These districts were selected based on program intensity, remoteness, and urban/rural nature. Number of trained CHWs was used as a proxy for program intensity. Remoteness was measured as the distance from Morogoro town, or distance from an urban center. A breakdown of how the districts were selected is shown in Table 1 below.

**Table 1:** Criteria for district selection for conducting Focus Group Discussions in the Morogoro region of Tanzania

District	Program intensity^a^	Remoteness	Urban/rural
Ulanga	Low intensity	Furthest	Rural
Mvomero	High intensity	Closest	Rural
KIlosa/ Gairo	Medium intensity	Intermediate	Urban

^a^Where number of CHWs in the district used as a proxy for program intensity

To promote diversity of experience, researchers selected two contrasting locations in each district. Selection of the research sites was based on accessibility by road, proximity to a town, and phone network coverage.

### Study design

This qualitative study employed semi-structured focus group discussions, where facilitators used discussion guides to steer conversations. Discussion guides, developed specifically for this study, consisted of a broad set of topical questions developed to meet the study aim (Additional files 1 and 2). Additionally, probes were included in the guide to elicit more detailed responses for each question. Facilitators were trained to ensure all questions were asked, however, they had the discretion on the order in which they were asked, given the direction and flow of the discussion. Facilities were also trained to probe for clarity, and/or where novel/unexpected responses arose. Focus groups with CHWs explored barriers in identifying pregnant women in the community and the challenges CHWs face in dispensing MNCH services and referring women to health facilities. Focus groups with RDWs explored perception, acceptance, and demand for CHW services.

### Selection of study participants

Two respondent groups were eligible for the study: CHWs who received training from the MoHCDGEC under the integrated MNCH program, and RDWs, the beneficiaries of MNCH services. We selected CHWs from a list provided by the Council Health Management Team (CHMT) under the MoHCDGEC. The list provided through the CHMT was comprehensive and listed all CHWs trained through the MAISHA program. The CHMT and implementing partner, Jhpiego, kept accurate program records to facilitate supportive supervision and for monitoring the program. We then purposively selected CHWs at each research site in order to reflect equal representation in terms of sex, and variation in length of employment, age, and educational attainment. All participants, however, shared the fact that they were selected by their village to receive CHW training.

We identified RDWs from CHW registers to ensure participants had previously interacted with community health providers. To gain nuanced insights of the RDW-CHW relationship, RDW were selected to represent variation and diversity in: 1) the number of visits they received from a CHW; 2) the timing of visit (pre or post-delivery); and 3) the distance they lived relative to the nearest health facility.

### Data collection techniques

Following three days of training, including a pilot test and revision of focus group discussion guide, three graduate research assistants, who speak fluent Kiswahili, and one researcher/supervisor collected data. A total of ten focus groups were conducted (five with CHWs and another five with RDW). In total, 34 CHWs and 34 RDW participated in the discussions in five selected locations. Each focus group had between six and eight participants and the discussions lasted one to two hours. Focus groups were held in the village offices or special rooms provided by the health facilities.

### Data analysis

Data analysis began in the field through daily debriefing sessions between the third author and the research assistants [17]. During these meetings, the research team discussed emerging themes, areas for further investigation and topics of saturation. An endpoint debriefing meeting was held between the field staff and researchers. The meeting offered a platform where data was triangulated across different respondent groups as well as between similar respondents from different locations.

All focus group discussions were digitally recorded after obtaining the participants’ consent, checked for quality and later transcribed in *Kiswahili*. These transcripts were then translated into English and a thematic analytical approach was adopted to evaluate the data and search for emerging themes that correlate to the concepts under investigation. The analysis involved a systematic review of all transcripts, enumerating themes of interest and including the most common themes for analysis.

### Ethical issues

Ethics approval was obtained from the Muhimbili University of Health and Allied Sciences and Johns Hopkins University institutional review boards. Further, local government structures granted clearance for this study at the Morogoro regional level, as well as through the Ulanga, Mvomero, and Kilosa/Gairo district administrations. Ward and village government leaders were also consulted prior to starting the study. Focus group discussions were conducted only after informed written consent was obtained from study participants. Participants were also informed of their right to withdraw from the study at any time. All the discussions were digitally recorded and manually recorded in notebooks with the permission of study participants.

## Results

Table 2 provides a summary of the demographic information of both groups who participated in focus group discussions. There was a relatively even number of male and female CHWs sampled. Fifty nine percent of CHWs were single and 56% had no children of their own. Of the RDWs who participated, 85% were married, and 53% had three or more children. Fifty six percent of RDWs were older than the majority of participating CHWs. Upon analysis three main themes emerged.

**Table 2:** Demographic characteristics of participants

Characteristics	CHWs		Women	
	Number (*n* = 34)	%	Number (n = 34)	%
Sex				
Male	18	53		
Female	16	47	34	100
Marital status				
Married	13	38	29	85
Single	20	59	5	15
Widow	1	3	–	–
Age (years)				
18–24	20	59	15	44
25–34	11	32	15	44
35–45	3	9	4	12
Level of Education				
None	–	–	9	26
Started primary school	–	–	2	6
Completed Primary school	4	12	19	56
Started secondary school	2	6	3	9
Completed Secondary school	28	82	1	3
Parity				
0	19	56	3	9
1–2	9	26	13	38
3 and Above	6	18	18	53
Work experience as CHW (in months)				
1–5	10	29		
6–10	24	71		

### CHW methods for identification of pregnant women

Community Health Workers reported several means by which they identified pregnant women in their communities. Their involvement in collecting information for the national census required them to ask households direct questions about pregnancy. This direct questioning was often the first way CHWs found out about pregnancies in their communities. Data collection for the census did cause some skepticism of CHWs in the community, however, and thus was not a means by which CHWs identified all pregnancies.

Community Health Workers identified most pregnant women through other community members. By making frequent household visits, CHWs were able to build relationships within the community. They recounted that these community members would inform CHWs if their neighbors and/or friends were pregnant. Similarly, CHWs who had strong relationships with local health facilities would share information about pregnant community members. In this case, CHWs would inform health facilities of women they had already made referrals for, and the health facility staff would inform CHWs of those who had already received services from them.

While household visits were highlighted as the primary mode of identifying pregnant women in their communities, CHWs reported frequent travel came at a personal cost. Traversing large geographical areas through difficult terrain, either by foot or bicycle, required time and physical exertion. As a volunteer cadre, CHWs were required to bear the financial costs of mending personal, donated, or hired bicycles were they to fall into disrepair as a result of their work. To facilitate travel across the “*long distances [they] travel”* CHWs recommended travel reimbursement from the local government and implementing partners:

*“They should think of giving us transport because some of us are coming from far away and we do not have transport. We hire bicycle to visit households but we don’t get anything in return”* (FGD, CHWs).

### Factors that affect community trust

Both CHWs and RDW reported several factors that lead the community to either trust or mistrust the CHWs. The former reported that the more familiar they were with community members, the quicker they were to trust the CHW, and thus disclose their pregnancies earlier:

“*When you visit women at their homes for the first they are very difficult to cooperate but when you visit them for the second and third times, they become free to talk and give you information that they could not give in the first visit.”* (FGD, CHWs).

This familiarity resulted from making frequent household visits, as well as being originally from the community.

Whether the community was familiar with CHWs or not, the demographics of the CHW also impacted how they could interact with RDW. Among RDW, the age of the CHW impacted how much information they would divulge about their pregnancy:

*“If she [CHW] is very young, I disclose some information and keep some, when an old one [CHW] visits me, I will tell her all problems of my pregnancy.”* (FGD, RDW).

Age of the CHW is significant in this study, as over 50% of RDW in the focus groups were older than the participating CHWs (Table 2). The marital status of the CHW also affected how the RDW thought of them. If a CHW was not married and didn’t have children of their own, the RDW did not believe that they would be able to inform them about their health:

*“CHW should be married and have children … What is she [female CHW] going to teach me? If she is not married and does not have children?”* (FGD, Female CHW).

Furthermore, RDWs worried about the impact that discussing their pregnancy complications would have on the young and unmarried CHWs:

*“If she (CHW) is very young and you tell her complications of pregnancy, you will cause her to fear being pregnant because she has never being pregnant.”* (FGD, RDW).

Several women in the focus groups reported positive experiences from disclosing their pregnancies to CHWs and receiving referrals to facility-based ANC services:

*“If a CHW writes a letter for you to take it to the health facility, once you reach there [health facility], you will easily get services.” (*FDG, RDW).

*“In my previous pregnancies, I experienced some problems and I was just tolerating [the discomfort]. A CHW came to my home and told me that if I am suffering from any illnesses, I should go and take referral letter from her and go to the health facility. Since then I go to the health facility and take some medication. I appreciate that they [CHWs] have good advice.” (*FGD, RDW).

Women also discussed how much they appreciated the health education and birth preparedness training that CHWs delivered:

*“This is my fourth pregnancy. First of all, I thank [the CHW] because I have never seen such a thing like birth preparedness in all my previous three pregnancies. Things like preparing clothes for the child like socks and hats, never! But I appreciate the CHW’s visit, it has woken me in terms of birth preparedness.”* (FGD, RDW).

Community Health Workers reported that trust for them spread most quickly within the community by positive word-of-mouth for their services among pregnant women.

### Reasons for early pregnancy concealment

Receiving referrals from CHWs for ANC services in the first trimester of pregnancy is essential to ensure that the ANC services are effective. Women described in focus groups why they might conceal their pregnancy from a CHW until it is farther along, often after the first trimester has passed. One woman explained that she may not know if she is pregnant until she is a few months along in the pregnancy:

*“Sometime I can stay for two months without having menstruation period, then I get it in the third month .... That is why I need to stay until three months to be sure if it is pregnancy before telling anyone.” (*FGD, RDW).

Community Health Workers further rationalized the norm of women waiting a few months before disclosing a pregnancy to a CHW due to fear of the pregnancy failing:

*“A woman told me that she cannot disclose that she is pregnant when the pregnancy is one month old because it can be aborted.”* (FGD, CHW).

*“They themselves, say they can be bewitched and they may suffer miscarriage if they disclose pregnancy in its early stage.”* (FGD, CHW).

The above phenomena are enumerated in Table 3, as the majority of women, regardless of their parity, attended their first ANC visit during the third and fourth months of gestation. Finally, women explained that CHWs are not typically the first people they are concerned with notifying about their pregnancy, further explaining this delay:

*“If I think that I am pregnant, the first person to tell is my husband, the second is my mother and the third person is my mother in law. Then I visit the health facility to inform CHW who visited me at home.”* (FGD, RDW).

**Table 3:** Percent of women attending first ANC visit by parity by gestational age

Parity	1	2–3	4–5	6+	Total
ANC 1 visit, gestational age (mos)	(%) (*n* = 401)	(%) (*n* = 714)	(%) (*n* = 452)	(%) (*n* = 324)	(%) (*N* = 1891)
1	0.75	0.00	0.00	0.31	0.21
2	4.99	3.22	4.65	4.01	4.07
3	31.17	34.87	34.29	28.4	32.84
4	32.92	33.47	28.98	33.95	32.36
5	16.71	18.91	19.25	19.14	18.56
6	10.22	6.86	10.40	10.49	9.04
7	2.24	2.24	1.99	3.40	2.38
8	1.00	0.42	0.44	0.31	0.53

## Discussion

**Fig. 1:**
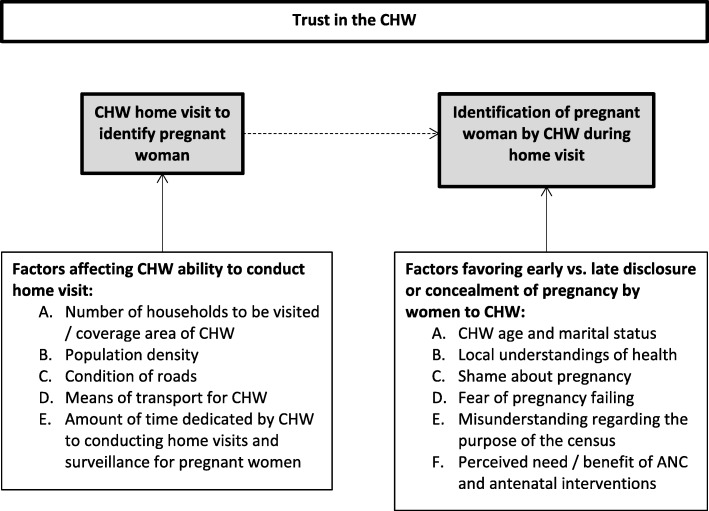
Factors that impact whether a woman will disclose her pregnancy early, late, or conceal it

Focus group discussions revealed several factors that affect CHWs’ ability to identify pregnant women early in their pregnancies during home visits. Figure 1 illustrates how these factors interact, leading a woman to either disclose, conceal, or delay disclosing her pregnancy to a CHW.

### Home visits to identify pregnant women

In focus groups, CHWs reported that through relationships built from frequent household visits, community members were more likely to be open about their health, including their pregnancies, if they were familiar with the CHW. Further, they would be more willing to help the CHW, informing them of pregnancies in the community. For this reason, CHWs who were originally from the community in which they were working had more immediate success identifying pregnant women than those who were placed there. Those CHWs who conducted frequent home visits most successfully built rapport with the community. Pregnant women would often disclose their pregnancies to CHWs during these visits as well, as they occurred in familiar and confidential settings.

As listed in Fig. 1, there are several environmental factors that impact CHWs ability to conduct home visits frequently enough to be effective [18]. They reported that the more households they had to visit, the longer it took them to gain rapport within communities, as they weren’t able to visit each individual household frequently enough. Rural areas posed a challenge to CHWs, as they often have low population density, resulting in more of the CHWs’ time being used to travel between households than in actual visits. In focus groups, CHWs described the difficulties they faced navigating rural roads to get to households in their communities. These challenges were reported to be seasonal, as roads were often flooded and impassable during the rainy seasons. The CHWs also reported that the lack of a reliably effective means of transport limited their ability to perform household visits more than any other factor. Given the volunteer nature of the CHW position, how much time devoted to home visits is at the discretion of the CHW.

### Factors favoring early versus late disclosure or concealment of pregnancy by women to CHW

Ideally, household visits would result in the woman disclosing her pregnancy to the CHW early in its course. Figure 1 outlines this relationship with household visits and factors RDWs identified as important in focus groups to determining when and if they would disclose their pregnancy to a CHW. Of note, is the idea that trust of the CHW is key for CHWs to succeed in identifying the pregnant woman, and when in the course of pregnancy, they will do so. This concept will be further addressed later in the discussion.

Many RDWs in focus groups emphasized the importance of the CHW’s age and marital status to their decision to disclose their pregnancy to them. Not wanting to scare the CHW from having her own child, and skepticism of the amount of wisdom the CHW had were both mentioned as reasons the RDW would attempt to conceal their pregnancies from an unmarried or young CHW. Further, older RDW demonstrated more uncertainty towards younger CHWs, especially if they already had several children of their own. This relates to prior research done on health seeking behavior among pregnant women. Age and marital status of the woman has been found to contribute to a woman’s decision to seek care in general [18].

Another finding was that RDW would often hesitate to disclose their pregnancies for fear of harm coming to their unborn child. Several locally held beliefs about the consequences of disclosing a pregnancy too early were reported in focus groups both by CHWs and RDW. Women who are pregnant out of wedlock can be far more reluctant to disclose their pregnancies. In these situations, women are fearful that the rest of the community will find out about their pregnancy if they disclose it to a CHW. This idea is supported by other studies on pregnancy and concealment, discussing social consequences of revealing pregnancy [11]. In this case, there is a lack of trust in the confidentiality of their interaction with the CHW. Women expressed that they would often wait until later in their pregnancies to disclose it to a CHW due to the uncertainty that it would last. This also reflects reports in the literature about women being secretive about their pregnancies until they are certain it will last to avoid the embarrassment of a failed pregnancy [11]. Often this would mean that they would wait until the beginning of the second trimester to disclose their pregnancy to a health worker, forgoing several ANC services that are essential to receive during the first trimester.

While in some cases the census assisted the CHWs with identifying pregnant women due to the direct nature of the questions; in most cases communities were skeptical of the census, and thus chose to conceal their pregnancies from the CHWs when asked during data collection. This had a lasting impact for the CHWs as well, as CHWs reported that this would sometimes create a stigma against them in the community beyond the period of data collection.

Finally, the woman’s choice to disclose her pregnancy, when to do so, or conceal it altogether, was ultimately determined by her perception of how much she needed the services that CHWs referred women to, and how much she might benefit from them. Gabrysch and Campbell wrote about this phenomenon, explaining that, if the woman understood the importance of the ANC services CHWs referred them to, and the health information they provided, they were more likely to reveal their pregnancies to CHWs early on [19]. In a study of the barriers to quality maternal healthcare in Tanzania, authors discovered a lack of availability of adequate resources necessary to assist in a healthy delivery in health facilities. Such resources extended to human resources, as health facility workers were often over-worked resulting in disrespectful and/or abusive treatment for 19–28% of women seeking care at health facilities [20]. Given these conditions, it seems likely that women would be hesitant to disclose their pregnancy to someone they perceive as a part of this system. Furthermore, women weigh this knowledge against their perceived level of social risk to make a decision to conceal, delay disclosure, or disclose her pregnancy [14].

### Levels of trust impacting women’s decision to disclose their pregnancies

Fig. 1 illustrates the over-arching nature of the impact of the concept of trust of the CHW on whether a woman will disclose her pregnancy early, late, or conceal it. Whether or not the community and individuals trust the CHW impacts whether or not a woman will disclose her pregnancy, when she will do so, and how she will receive recommendations and referrals from the CHW. These dynamics were a popular theme through focus group discussions with both CHWs and RDW.

Several levels of trust were identified in focus groups that impact a woman’s decision to disclose her pregnancy early on; starting with community trust, then family trust, and finally the woman’s trust of the CHW. On the community level, CHWs reported that trust was often built by positive word-of-mouth. In the case of CHWs, it was found that an understanding of CHWs’ training and purpose in the community often lead the first set of individuals to trust and accept the CHW, and with time, more and more households came to accept the CHW.

Ultimately, it is the level of trust an individual woman has for the CHW that determines her decision to either conceal or disclose her pregnancy, and when to do so. Furthermore, the woman’s trust of the CHW impacts the efficacy of every interaction the CHW has with the woman. For example, the amount of trust a woman has for a CHW impacts how effective a home visit can be. If the woman does not trust that the CHW will give her information that will be helpful to her, and further, if the woman does not trust that the CHW won’t hurt her in some way, she will be less likely to disclose her pregnancy to the CHW during that home visit.

Trust can also affect how a woman perceives the factors favoring early vs. late disclosure or concealment of a pregnancy. A woman who already trusts the CHW will be less concerned about if a CHW is young or single. Further, a woman won’t be as concerned about shame from the community about her pregnancy, as she will trust that her interaction with the CHW is confidential. Trust can also impact a woman’s perceived benefit from the services and referrals a CHW provides. If the woman trusts the CHW, she will have a more positive perception of their services.

### Study limitations

This study would have benefitted from ensuring that both women who did and did not disclose their pregnancies early in gestation were included in focus groups. This would have ensured that both perspectives were equally represented in the data. This information, however, was difficult to obtain prior to conducting the focus groups. To mitigate this limitation, we asked participants to share experiences they have seen or heard of in their homes or communities. Second, focus groups tend to elicit the normative behavior of communities, rather than individual factors that affect disclosure. The addition of one-on-one semi-structured interviews would have allowed for these factors to be discovered. This work, however, gave insight into some challenges of early pregnancy identification that future research can build upon. Finally, qualitative inquiry using focus groups is vulnerable to social desirability bias, resulting in some respondents possibly over-reporting or under-reporting the benefits or challenges of pregnancy identification in the CHW program. Several relevant cultural factors, such as religion, might affect the quality of information collected in the study. To counter this, the research team established rapport with participants, created a welcoming environment, and reassured them of confidentiality. Despite such limitations, this study has generated valuable insights about factors affecting early identification of pregnant women by community health workers in the study area.

## Conclusions

Several factors were identified that affected CHWs’ ability to identify pregnant women early in pregnancy. Those that inhibited identification include the woman’s knowledge of her pregnancy, CHWs being a low priority to notify, and skepticism of the CHW because of age and marital status. Women’s perceived need and benefit of the services provided by the CHWs, as well as the amount of trust women have for CHWs, were factors that had a broader impact on the CHW-RDW relationship, and CHWs’ ability to identify pregnant women.

More studies need to be done to investigate the optimal work-load for CHWs. Further, research needs to be done into why pregnant women might conceal their pregnancies from health workers. More research might be conducted on diffusion of innovations, such as early identification of pregnancies and community-level interventions. This can enhance CHW training through a deeper understanding of community dynamics. Finally, a quantitative investigation into the identified factors facilitating and hindering pregnancy identification in the CHW program would provide further insight into the situation. Such research is necessary to glean better information to develop strategies to identify women early in gestation to make timely referrals to facility-based ANC and improve pregnancy outcomes. Further engagement on the community level is also recommended to reevaluate how CHWs work in Morogoro.

## Additional files

Additional file 1: Instrument #D2a: Discussion guide for maternal and child health CHWs. (PDF 517kb)

Additional file 2: Instrument #D2b: Discussion guide for Mothers/CHW Clients. (PDF 819kb)

## Data Availability

The datasets analysed during the current study are available from the corresponding author and Principal Investigator of this study on reasonable request.

## Abbreviations

ANC: Antenatal Care
CHW: Community Health Worker
MNCH: Maternal, Newborn, and Child Health
MoHCDGEC: Ministry of Health, Community Development, Gender, Elderly, and Children
RDW: Recently Delivered Woman
