# Supplementary material for: Factors affecting early identification of pregnant women by community health workers in Morogoro, Tanzania
Source: BMC Public Health. 2019 Jul 8;19:895. doi: 10.1186/s12889-019-7179-1 (PMC6615291; doi:10.1186/s12889-019-7179-1)
Supplement: Supplementary file 1 — Instrument #D2a: Discussion guide for maternal and child health CHWs. (PDF 517kb) [file 12889_2019_7179_MOESM1_ESM.pdf]

Instrument #D2a: Discussion guide for maternal and child health CHWs

Muhimbili University of Health and Allied Sciences (MUHAS)
Johns Hopkins Bloomberg School of Public Health
Evaluation of Integrated Maternal and Newborn Health Care Program in Morogoro Region, Tanzania
JHSPH IRB 3296 – MUHAS IRB
Version Date: 16 July 2013

What is the objective of this study? This guide provides questions to facilitate focus group discussions with community health workers who are providing maternal, newborn and child health services in the community. The objective of the focus group discussion is to assess the facilitating factors and barriers for community health worker programs in Tanzania.

INSTRUCTIONS TO THE FACILITATOR

1. Ensure that the discussion is taking place in a private setting with seating for all participants.
2. Ensure that all participants have number placard, which is visible to the facilitator and the note taker.
3. Before asking the first question on the discussion guide, read the introduction script and answer any questions that participants may have.
4. As a facilitator, it is important to encourage all participants to contribute to the discussion for each topic. Prompts to encourage others to participate include:
    a. Does everyone else agree?
    b. What do you on this side of the circle think?
    c. Has anyone had a different experience?

5. As appropriate, ask follow up questions to participants to better understand their opinions. Possible follow up probes include:
    a. Why do you think that?
    b. Can you tell me an example?
    c. Can you tell me why a person might think that way or perform that action?

Introduction

My name is ___________________________ and I work for Muhimbili University in Dar es Salaam. Today I would like to hear your opinions about your work as a community health worker .You were asked to participate in this discussion because you have been providing health services to pregnant women, postpartum mothers and children in your community. We are interested in your opinions about the community health worker program so that we can make recommendations for improving the program.

In the discussion we will be interested to hear your opinions. As such there are no right or wrong answers because you are only expected to share your experiences. We want all participants to feel comfortable to contribute, so please do not share the content of the discussion with others. Your name will not be reported as a participant in the study, and during the discussion we will address you only by the number that you received.

Before we start do you have any question?

Discussion Questions

Implementation of the MNH Program at the Community Level

1. Now I would like to ask about the home visits that you are making to pregnant women, postpartum women, and children, and how the families in the communities where you worked received the home visits.

- In general, what do you think is the community's opinion about this CHW program?
- What do families like about the home visits? What don't they like about them?
    - Do they prefer pregnancy, postpartum, or childhood visits?
    - Have you received any negative feedback or refusals from women or their families? if you have, what are the reasons women give negative feedback/refuse you to visit them?

2. How do you learn about, and identify pregnant women in the community?
    - How early will women tell the CHW about their pregnancy? Is it the same for friends and neighbors?

3. Do you know what the recommended number of home visits by a CHW is:
    - During pregnancy? How many say 1 time? How many say twice? How many say three times?
    - After the child is born? How many say 1 time? How many say twice? How many say three times?
4. Have you encountered any challenges in making home visits as frequently as the guidelines recommend?
    - Do you have enough time during the week to visit women?
    - Can you make visits within 24 hours of birth? Why or why not?

Support Systems

5. Now I would like to ask about the support you receive for your work as a CHW from the health facility.
    - How do the facility workers support you?
    - Are there any changes you would like to see in this support?
    - Do you ever receive supervision or visits from facility-based health workers? If yes, can you tell us what happens in such a visit?
    - Do you feel you need further training, coaching or supervision?
    - Are there any materials or equipment you need to better do your work?

6. We would also like to know about any support you receive from other community members.

- Are there any other community members who support you in your work as a CHW? If yes, who are they? How do they support you? In your opinion how could the community provide more support to you? In your opinion, should the community provide any financial support for your work? Why and how?
- Do the ward or village government officials provide any support to you for your work as a CHW?
- Is there a health committee in your area? Do they help you in any way with your work?
- Do you have any role in this committee? What do you do, or how do you help them with their work?

Ability to work effectively with both men and women

7. In your opinion does the age, gender, level of education, marital status or tribe make a difference in their ability to counsel women and families?
   - How is the experience of a female CHW different from the experience of a male CHW?
   - How is the experience of an older CHW different from that of a younger CHW?
   - How does the experience of a CHW who was born in this area differ from the experience of a CHW who migrated to this area as an adult?

8. Have you ever referred a woman or child to a health facility for care?
   - Describe what happened?
   - What barriers did you face in making a referral?
   - Did you accompany the family to the health facility? Did that help? What did you do when you arrived at the health facility to assist the family?
   - Did you hear back what happened after you made the referral?

HIV Policies

9. Now I'd like to ask your opinion about the role of CHWs in supporting people with HIV/AIDS in your community.
   - Are there home-based care workers in your community? What is their role in the community? What does the community think about them?
   - What would you think about adding visits to HIV-positive people to the work that you currently do?
     - What would be the good things about it?
     - What would be the bad things about it?

10. Tanzania is currently changing the services for HIV-positive pregnant women. Currently, women who test positive for HIV during antenatal care are offered treatment to prevent the HIV virus from being transmitted to the baby.

Starting this year, all women who test positive for HIV during pregnancy will be offered drugs for the rest of their lives to prevent the HIV virus from making them sick.

- What is your opinion about this change?

- What questions would you have for health providers about this change?
- Do you have any suggestions for how to implement this new policy?

Closing

11. In general, what are the things that you like about your work as a CHW? What are the things that you don't like?

12. Are there any suggestions you would like to communicate to the Ministry of Health and Social Welfare about improving this program?
