# Supplementary material for: Factors affecting early identification of pregnant women by community health workers in Morogoro, Tanzania
Source: BMC Public Health. 2019 Jul 8;19:895. doi: 10.1186/s12889-019-7179-1 (PMC6615291; doi:10.1186/s12889-019-7179-1)
Supplement: Supplementary file 2 — Instrument #D2b: Discussion guide for Mothers/CHW Clients. (PDF 819kb) [file 12889_2019_7179_MOESM2_ESM.pdf]

Instrument #D2b: Discussion guide for Mothers/CHW Clients

Muhimbili University of Health and Allied Sciences (MUHAS)<br>Johns Hopkins Bloomberg School of Public Health<br>Evaluation of Integrated Maternal and Newborn Health Care Program in Morogoro Region, Tanzania<br>JHSPH IRB 3296 – MUHAS IRB<br>Version Date: 16 July 2013

What is the objective of this study? This guide provides questions to facilitate focus group discussions with women who are receiving maternal and child health services from CHWs in their communities. The objective of the focus group discussion is to assess the facilitating factors and barriers for community health worker programs in Tanzania.

INSTRUCTIONS TO THE FACILITATOR

1. Ensure that the discussion is taking place in a private setting with seating for all participants.
2. Ensure that all participants have number placard, which is visible to the facilitator and the note taker.
3. Before asking the first question on the discussion guide, read the introduction script and answer any questions that participants may have.
4. As a facilitator, it is important to encourage all participants to contribute to the discussion for each topic. Prompts to encourage others to participate include:
    a. Does everyone else agree?
    b. What do you on this side of the circle think?
    c. Has anyone had a different experience?
5. As appropriate, ask follow up questions to participants to better understand their opinions. Possible follow up probes include:
    a. Why do you think that?
    b. Can you tell me an example?
    c. Can you tell me why a person might think that way or perform that action?

Introduction

My name is ____________________________ and I work for Muhimbili University in Dar es Salaam. Today I would like to hear your opinions about maternal and newborn health services in the communities where you live. You were asked to participate in this discussion because you have received visits from the community health worker in your area during your pregnancy and/or after you delivered your baby. We are interested in your opinions about the community health worker program so that we can make recommendations for improving the program.

In the discussion we will be interested in hearing your opinions. As such there are no right or wrong answers because you are only expected to share your experiences. We

want all participants to feel comfortable to contribute, so please do not share the content of the discussion with others. Your name will not be reported as a participant in the study, and during the discussion we will address you only by the number that you received.

Before we start do you have any question?

Discussion Questions

Introduction

1. Going around in a circle, can each of you please tell me how old your most recent baby is, and how many times you were visited by a community health worker during and after your pregnancy?

General Impressions of CHWs

2. Now I would like to ask about the visits that you received from CHWs during your pregnancy.
   - How many of you received a visit from a CHW during your pregnancy (ask for a show of hands)?
   - What did you like about the CHW's visit?
   - What didn't you like about the CHW's visit?
   - Is there anything you wish the CHW would do differently? If yes what are they?

3. How do CHWs learn which women in the community are pregnant? What do they do to find this out?

4. How do you think CHWs should learn about pregnant women in the community?
   - How early in pregnancy do you think CHWs should visit women?
   - How early will women tell the CHW about their pregnancy?
   - How early will women tell their friends and neighbors about their pregnancy?

5. Now I would like to talk about the visits that you received from CHWs after your pregnancy.
   - How many of you received a visit from a CHW after you delivered your baby (ask for a show of hands)?
   - What did you like about the CHW's visit after delivery?
   - What didn't you like about the CHW's visit?
   - How often should CHWs visit women with young children? Why?
   - Is there anything you wish the CHW would do differently for mothers and children? If yes, what are they?

Linkages between CHWs and Health facilities

6. Did the visits from the CHW have any effect on your use of facility services during pregnancy? If yes, how? Why?
   - Did information given by the CHW help you to plan postpartum visits for your health after delivery?

- Did you receive any visits from the CHW? Did these visits encourage you to visit a health facility to receive postpartum care there? If yes, how?

7. What do you think is the relationship between CHWs in your community and the dispensary or health center?
   - How often do CHWs visit the facility or talk with facility providers? Is this the right amount of visits?
   - What information should the CHWs and health providers share with each other?

Relationships between CHWs and women

8. Does who the CHW is, what kind of a person the CHW is, make a difference to you or other women in the community when receiving counseling about maternal and child health issues?
   - Why or why not?
   - Does it matter if the CHW is a man or a woman?
   - Has anyone had experiences with a male CHW? What did you think?
   - Are there differences between male and female CHWs? What are they?
   - How do your husbands/partners react to CHWs visiting you at home? Does this reaction differ by the sex of the CHW?
   - Does it matter if the CHW is old or young?
   - Does it matter if the CHW is from this area or from another part of the country originally?

HIV Policies

9. Now I'd like to ask your opinion about the health services for women who test positive for HIV during pregnancy. Currently, women who test positive for HIV during antenatal care are offered treatment to prevent the HIV virus from being transmitted to the baby.
   - What do you think about these services? Is it good for HIV-positive women?
   - Is there anything that you think women don't like about these services?

10. Tanzania is currently changing the services for HIV-positive pregnant women. Starting this year, all women who test positive for HIV during pregnancy will be offered drugs for the rest of their lives to prevent the HIV virus from making them sick
    - What is your opinion about this change?
    - What questions would you have for health providers about this change?
